# Complete Genome Sequence of Lactococcus cremoris Strain 7-1, a Lactic Acid Bacterium Isolated from a Traditional Mongolian Milk Product Possessing Mucin-Adhesive Ability

**DOI:** 10.1128/mra.00143-22

**Published:** 2022-04-04

**Authors:** Hirotoshi Sushida, Hiroaki Sakai, Naoko Moriya, Kazuma Nakano, Noriko Ashimine, Maiko Minami, Hinako Tamotsu, Tetsuhiro Nakanishi, Shun Ohki, Kuniko Teruya, Takashi Hirano, Seishi Yamasaki, Chise Suzuki, Kazuhito Satou, Hiromi Kimoto-Nira

**Affiliations:** a Institute of Food Research, NARO, Tsukuba, Ibaraki, Japan; b Advanced Analysis Center, NARO, Tsukuba, Ibaraki, Japan; c Institute of Livestock and Grassland Science, NARO, Tsukuba, Ibaraki, Japan; d Okinawa Institute of Advanced Sciences, Uruma, Okinawa, Japan; e Crop, Livestock, and Environment Division, JIRCAS, Tsukuba, Japan; University of Maryland School of Medicine

## Abstract

We report the complete genome sequence of Lactococcus cremoris strain 7-1, which was isolated from urum, a traditional Mongolian milk product. Strain 7-1 adhered to porcine gastric mucin in a carbon source-dependent manner. The genome consists of a circular chromosome (2,557,589 bp; GC content, 35.7%) and two circular plasmids.

## ANNOUNCEMENT

The adhesion of bacterial cells to the mucosal surface is a primary property of probiotics (1). Lactic acid bacteria (LAB) are the most widely recognized probiotic bacteria. Previously, the mucin-adhesive strains were screened from LAB strains isolated from Mongolian milk products (2). Subsequent analysis revealed that Lactococcus cremoris strain 7-1 exhibited unique mucin-binding activity, which was altered depending on the carbon source (2). Here, we report the complete genome sequence of strain 7-1 to elucidate the regulatory mechanisms of the carbon source-dependent probiotic properties.

Strain 7-1 was isolated from urum, a traditional Mongolian clotted cream, and stored as glycerol stock at −80°C. Strain 7-1 was propagated to the early log phase in MRS medium at 30°C. The genomic DNA was extracted as previously described (3) and purified using a PowerClean DNA cleanup kit. A 20-kb library was constructed without size selection using a g-TUBE device (Covaris) for P6-C4 chemistry shearing following the standard protocol recommended by PacBio. Eight single-molecule real-time (SMRT) cells (240-min movie each) were used for sequencing on the PacBio RS II platform. We assembled 396.0 Mbp of subreads, which corresponds to 158.4-fold coverage of the estimated genome size (2.5 Mbp), using Canu v. 1.7 (4). The quality control and trimming were also performed using Canu, which produced 14,009 reads with a mean length of 6,541 bp. The resulting contigs, which were predicted to be circular by Canu, were edited as starting from the *dnaA* sequence using Circlator (5). Default parameters were used for all software unless otherwise specified.

The genome of strain 7-1 consisted of one chromosome (2,557,589 bp; GC content, 35.7%) and two plasmids, pLC71A and pLC71B (98,383 bp and 31,483 bp; GC content, 35.4% and 34.9%, respectively). The number of coding sequences predicted by the DFAST pipeline was 2,598 for the chromosome and 88 and 32 for pLC71A and pLC71B, respectively. The sequence alignments were performed using BLAST with default parameters. The chromosome contains two putative mucin-binding proteins. The protein sequence of Llc71_20560 exhibited 76% identity with the elongation factor Tu (EF-Tu) of Lactobacillus johnsonii NCC 533 (NCBI reference sequence WP_004894320.1), and Llc71_25660 had 61.88% identity with the type I glyceraldehyde-3-phosphate dehydrogenase (GAPDH) of Lactiplantibacillus plantarum WCFS1 (NCBI reference sequence WP_003643974.1). EF-Tu and GAPDH have been reported as pH-dependent sulfated-galactosylceramide-binding proteins (6, 7) and human A and B blood group antigens (8), respectively. The predicted functions and carbon source-dependent regulation of these proteins will be investigated in future studies.

A transcriptional analysis using various carbohydrates and *in vitro* characterization of putative functional proteins of strain 7-1 may provide critical insights into the mechanism of the variable mucin-binding ability.

### Data availability.

The genome sequences of *L. cremoris* strain 7-1 were deposited in DDBJ/GenBank under the accession numbers AP025520 (chromosome), AP025521 (pLC71A), and AP025522 (pLC71B). The DDBJ Sequence Read Archive (DRA) accession number is DRA013132. This paper describes the first version of the genome.
